# Piloting of a Decision Aid for Recurrent Tonsillitis

**DOI:** 10.1111/coa.14278

**Published:** 2025-01-16

**Authors:** Callum Hill, Kim Ah‐See, Helen Moffat

**Affiliations:** ^1^ The University of Aberdeen NHS Grampian, Aberdeen Royal Infirmary Aberdeen Scotland UK; ^2^ Ear, Nose and Throat Department NHS Grampian, Aberdeen Royal Infirmary Aberdeen Scotland UK; ^3^ Clinical Psychology Department NHS Grampian, Aberdeen Royal Infirmary Aberdeen Scotland UK

**Keywords:** decision aid, sore throat, surgery, tonsillectomy, tonsillitis

## Abstract

**Objective:**

Currently, there is no adult‐specific decision aid (DA) to support decision‐making regarding recurrent tonsillitis. This study intends to address this gap by piloting a prototype DA.

**Design:**

Randomised clinical trial.

**Setting:**

Single centre trial at a tertiary otolaryngology department.

**Participants:**

Forty‐three patients were randomised to either the DA or Treatment as Usual (TAU) group.

**Main Outcome Measures:**

*Primary objective*: To measure how patients rate the quality of their decision‐making experience at the time of the decision and at follow‐up (SURE scale).

*Secondary objective*: The level of decisional satisfaction at the time of the decision and follow‐up, as well as to explore the numbers of people opting for surgery for each study condition (Shared tool and patient feedback).

**Results:**

*Quality*: This study demonstrates no statistically significant difference in how patients rate the quality of their treatment decision between DA and TAU, both at baseline (*p* = 0.553) and follow‐up (*p* = 0.062).

*Satisfaction*: This study showed a statistically significant level of decisional satisfaction at the time the decision was made for Qu2 of the Shared tool (*U* = 113, *p* = 0.026). No other significant difference was found between participants who received the DA and TAU.

**Conclusion:**

The DA is an acceptable and useful tool that could be incorporated into the pathway for recurrent tonsillitis, helping to eliminate physician implicit bias. However, preliminary qualitative evidence from this pilot study does not suggest that including the DA improves the quality of decision‐making.

**Trial Registration:** IRAS ID ‐ 230 362

**Summary**

- Decision aids can be useful in helping patients decide between surgical and non‐surgical interventions for recurrent tonsillitis in adults.
- Decision aids may be a useful tool in eliminating physician implicit bias.
- Decision aids do not demonstrate increased quality of decision‐making or decisional satisfaction versus treatment as usual in relation to the treatment of recurrent tonsillitis in adults.
- Decision aids may allow patients to discuss risks and benefits in greater detail with their surgeon if provided before their appointment.

## 1 Introduction

Early in our medical education, we are taught the importance of adopting a holistic, patient‐centred approach when treating patients. A critical aspect of this is being aware of patients' values and accommodating such values while considering healthcare options. However, before any decision can be made, informed consent is required. Three fundamental criteria must be met for informed consent: lack of coercion, competence and being adequately informed [1]. Unfortunately, patients can sometimes feel they lack autonomy in the decision‐making process regarding their treatment. Even with autonomy, a patient's decision‐making can be influenced by the clinical setting and how a health issue or treatment option is presented. Furthermore, a patient's decision may be based on their experiences or circumstances. Evidence indicates that when fully informed, patients choose different treatments [2]. However, a recent survey demonstrated that even when presented with different treatment options, only 64% of patients asked about the pros and cons of each treatment [3]. It is essential to acknowledge the significant discrepancies between patients' desires and healthcare professionals' perceptions of those desires. Thus, communication is vital in understanding a patient's values, the impact of the condition on the patient's life, and what treatment option may best support a positive change for the patient while also considering what is an acceptable risk.

Shared decision‐making (SDM) empowers patients in decision‐making [4]. SDM can be defined as a collaborative process between patient and clinician involving: (1) information exchange—identifying patient values, treatment options offered, risks and benefits discussed; (2) deliberation—patient preferences are considered against the risks and benefits; (3) implementation—treatment of choice undertaken [4]. Thus, the application of SDM allows patients to make an informed choice. To further assist this, support tools such as a decision aid (DA) can be utilised [5]. Such tools can provide a platform where SDN can be delivered, promoting collaborative communication around the various treatment options available and permitting greater unity between the patient and healthcare provider during the decision‐making process [6, 7, 8].

One area in which SDM and DAs may benefit patients is within adult tonsillectomy. In 1998, the Scottish Intercollegiate Guidance Network (Sign) introduced new guidelines to improve patient selection for tonsillectomy while reducing the potential harm from complications [9]. The new guidelines stipulated that to undergo a tonsillectomy, one should have experienced seven or more well‐documented, clinically significant episodes of tonsillectomy within a one‐year period, or five or more such episodes within the previous 2 years, or three or more such episodes within the previous 3 years [10]. As predicted, the number of tonsillectomies performed since then has reduced dramatically [9]. Furthermore, in 2000, there was a shift from multiple‐use to single‐use tonsillectomy instruments due to the theoretical risk of prion transmission from patient to patient [9]. The rising costs resulted in a further reduction in tonsillectomies being performed [10]. At the same time, retrospective studies demonstrated a significant rise in hospital admissions related to tonsillitis in patients who had not undergone tonsillectomy [11]. However, the increase in hospital admissions may offset the supposed savings by reducing the number of tonsillectomies performed. A Cochrane review demonstrated that although there were reasonable levels of evidence for tonsillectomy within the paediatric population, for adults, this was not the case [12]. This has since been challenged with the NATTINA trial, which has demonstrated that early tonsillectomy is both clinically and cost‐effective in adult recurrent tonsillitis [13].

Despite tonsillectomy being an effective approach for adult recurrent tonsillectomy, it may not be the most appropriate for each patient. It is important to remember that other aspects may influence a patient's decision, including, but not limited to, financial implications, work absences, and psychological and emotional impact [14].

This study aims to address this gap by implementing a novel DA. The goal is to highlight the pros and cons of each clinical management plan and incorporate patient values, supporting a more overt discussion between the patient and the clinician.

## 2 Objectives

### 2.1 Primary Objective

To measure how patients rate the quality of their decision‐making experience at the time of the decision and at follow‐up, either with a DA or in TAU (SURE scale).

### 2.2 Secondary Objective

To assess the level of decisional satisfaction patients feel at the time of the decision and at follow‐up and to explore the numbers of people opting for surgery from each study condition (Shared tool and patient feedback).

## 3 Methods

### 3.1 Study Design and Setting

**FIGURE 1:**
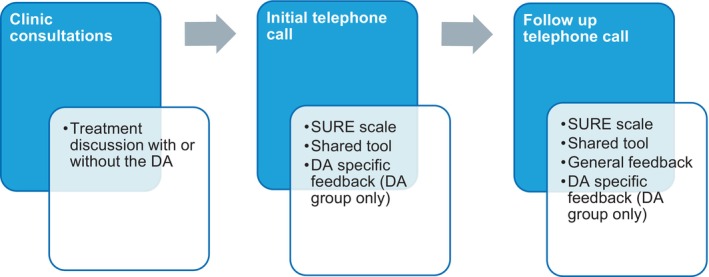
Study design.

Figure 1 outlines the study design: a single‐centre pilot randomised controlled trial investigating the effectiveness of a novel DA (shown in Figure S1) for treatment selection in adults experiencing recurrent tonsillitis. Having only a single ENT consultant limited the risk of clinician effect on results.

A draft DA for adults with tonsillitis was developed based on a review of the literature with input from ENT clinicians and psychologists. To determine its clinical utility, feedback on the draft DA was also sought from patients recently attending the ENT clinics for tonsillitis within NHS Grampian based on this feedback, some amendments were made to the draft to produce the final prototype DA used for the study (Figure S1).

### 3.2 Recruitment

All NHS Grampian patients currently under the care of ENT, undergoing treatment for recurrent tonsillitis, and not participating in any ongoing research trials were eligible. The ENT consultant involved in the trial identified and consented all participants. Forty‐three participants with recurrent tonsillitis were recruited to attend the ENT outpatient clinic. Exclusion criteria included patients under 16 years old and those not meeting the SIGN guidelines [15].

### 3.3 Randomisation

The NHS Research Ethics Committee approved the study protocol.

Participants were allocated to either the Treatment as Usual group (TAU) or the DA through alternative allocation to each group, DA (*n* = 22) or TAU (*n* = 21), by the ENT clinical administrator during the process of sending out clinic letters. Blinding was not possible as the ENT consultant utilised the DA during the discussion around treatment options with the participants of the DA group. However, participants were allocated study identification numbers to limit their responses being directly identifiable.

### 3.4 Intervention

Participants from both groups followed the same study procedure, except for receiving the DA or TAU, along with usual clinic correspondence and the study information sheet.

While attending the clinic, the consultant discussed the treatment options, either with or without the DA, according to the randomisation. A researcher planned to contact the participants by telephone within 1 week of the consultation to gather baseline measures. However, the average time for contact was 2 weeks later. During the phone call, the following questionnaires were utilised: Tonsillectomy outcome inventory, SURE scale and Shared tool. Furthermore, feedback on the DA was collected for those within the DA group [16].

**FIGURE 2:**
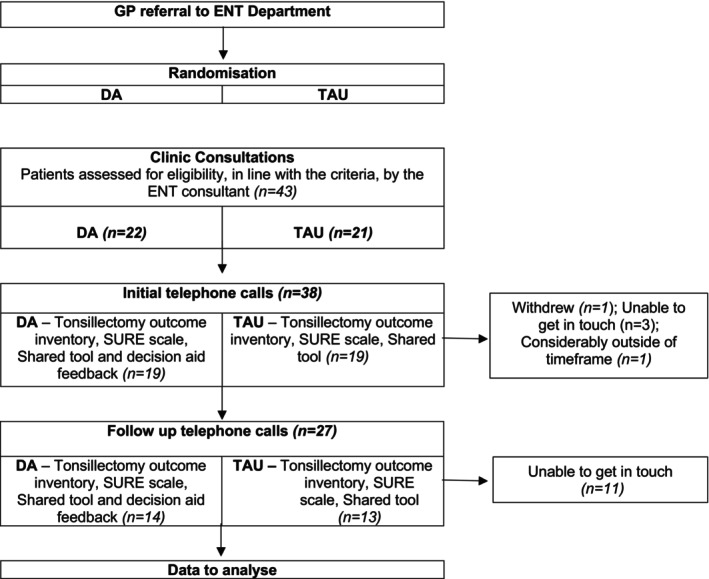
Flow diagram of the study.

Follow‐up data were collected at different time points, depending on whether the participant chose surgery or conservative treatment options. For both the DA and TAU groups, patients who chose surgery were due to be followed up at 1 month after their surgery date. However, the average follow‐up time was 1.5 months after the consultation. Patients from the DA and TAU groups who did not choose surgery were due to be followed up at 4 months after their initial clinic consultation, although the average was 4.6 months (Figure 2).

### 3.5 Outcomes

The primary objective of this study was to measure how patients randomised to either DA or TAU rate the quality of their decision‐making experience at the time of the decision and at follow‐up. This was measured using the SURE scale [17, 18]. A 4‐item rating measure with scores ranging from 0, extremely high decisional conflict, to 4, no decisional conflict.

Secondary to this outcome was assessing the level of decisional satisfaction the patients felt at the time of the decision and follow‐up. Decisional satisfaction was measured by the Shared tool [15]. A 4‐point scale measure contains eight items consisting of statements about decision‐making experiences, with responses ranging from agree strongly to disagree strongly. A further secondary outcome was to explore whether the DA influenced the number of people opting for surgery. Other outcomes of interest included the acceptability of the prototype aid, measured through participant feedback.

### 3.6 Statistical Analysis

Qualitative statistical analysis of scores at baseline and follow‐up was performed using the IBM SPSS 25 program. A Mann–Whitney *U* test was performed to determine if there was a difference in the degree by which a patient rates the quality of their treatment decision between participants who received the DA and those who did not. A Mann–Whitney *U* test was performed to determine if there was a difference in decisional satisfaction between participants who received the DA and those who did not. A chi‐square test of independence was performed to analyse any associations between the participant group and their treatment decision.

## 4 Results

This study demonstrated no statistically significant difference in the degree by which patients rated the quality of their treatment decision between DA and TAU, both at baseline, *p* = 0.553 and follow‐up, *p* = 0.062 (Table 1).

**TABLE 1:** A comparison in the degree by which patients rate the quality of their treatment decision when using a decision aid versus treatment as usual.

Group	Baseline SURE		Follow up SURE	
	Mean	*p*	Mean	*p*
DA	3.93 (*n* = 19)	0.553	4.00 (*n* = 14)	0.062
TAU	8.85 (*n* = 19)		3.38 (*n* = 13)	

Abbreviations: DA, decision aid; TAU, treatment as usual.

Table 2 revealed a statistically significant level of decisional satisfaction at the time the decision was made (baseline) for Qu2 of the Shared tool: “I felt the health professional thought one option was better for me than another” (Mann–Whitney *U*, *U* = 113, *p* = 0.026). No other significant difference was found at baseline or follow‐up between the participants who received the DA and those who did not.

**TABLE 2:** A comparison in the level of decisional satisfaction for those who received decisional aid versus treatment as usual at the baseline and follow‐up.

Shared tool item		Baseline (p)	Follow‐up (p)
		(*n* = 38)	(*n* = 27)
Qu1	The health professional talked about other options from the one we chose	0.612	0.402
Qu2	I felt the health professional thought one option was better for me than another	0.026	0.325
Qu3	I felt it was OK to choose an option that was different from the health professional's choice	0.111	0.519
Qu4	I felt the health professional gave me the support and advice I needed to make the best decision for me	0.881	0.375
Qu5	I was able to tell the health professional what was important to me about this decision	0.290	0.616
Qu6	I am clean about the benefits and risks of each options	0.102	0.166
Qu7	I am clear which benefits and risks matter most to me	0.628	0.076
Qu8	I am sure the option we chose is the best one for me	0.090	0.375

Abbreviations: DA, decision aid; TAU, treatment as usual.

Table 3 demonstrates a higher proportion of patients choosing surgery as a treatment option in the DA group (89.5%) versus the TAU group (63.2%). However, chi‐square analysis indicates that this proportional difference between the two groups is not statistically significant (chi‐square, *p* = 0.056).

**TABLE 3:** A comparison in treatment choice for those who received the decision aid versus those who received treatment as usual.

Group	Decision	
	Surgery	Non‐surgical
DA (*n* = 19)	17 (89.5%)	2 (10.5%)
TAU (*n* = 19)	12 (63.2%)	7 (36.8%)

*Note*: Chi‐square analysis—*p* value = 0.056.

Abbreviations: DA, decision aid; TAU, treatment as usual.

Feedback indicated that all participants within the DA group thought the pilot DA was a comprehensible, acceptable, feasible and desirable tool when discussing treatment options for recurrent tonsillitis.

## 5 Discussion

This study explored the use of a pilot DA within the treatment of recurrent tonsillitis and whether such a tool can assist the decision‐making process. The results demonstrated no statistically significant difference in the degree by which patients rate the quality of their treatment decision between DA and TAU, both at baseline (Mann Whitney *U*, *p* = 0.553) and follow‐up (Mann–Whitney *U*, *p* = 0.062) or decisional satisfaction. However, both the DA and TAU stated they had positive experiences. Therefore, the DA is an acceptable tool in the decision‐making process but does not demonstrate an increased quality of decision‐making or decisional satisfaction compared with TAU.

Although quantitative statistical analysis showed limited statistical significance in supporting the use of a DA, it was superior to TAU alone. Participant feedback from the DA group demonstrated that the pilot DA was easy to understand, providing usual information, and thus was an acceptable supplement to TAU alone. Furthermore, several participants reported feeling that the DA allowed them to assess the risks and benefits of each treatment option, giving them insight prior to the consultation with the consultant and allowing them to ask questions during the consultation. Interestingly, most of the TAU group believed the consultant provided enough information for them to choose. However, when one patient experienced a complication post‐surgery, they believed that although the risks had been outlined, they were “touched on bluntly.” Another patient from the TAU group felt they had too much information to process in the consultation, leaving them anxious and with questions unanswered. One such question the participant was particularly anxious about was the risks surrounding antibiotic use. The DA discusses the risks of antibiotic use, and thus, it may have resolved the anxieties of this participant. From these statements, it may be reasonable to suggest that the use of a DA in patients with increased anxiety or complications may help to resolve such issues. However, we cannot draw any conclusions about this topic due to the limitation of a small group of individuals experiencing such issues within this study. Further studies should be completed to explore the use of DAs within patients with anxiety, as well as patients who have experienced complications.

The feedback highlighted that participants believed the physician favoured non‐surgical treatment over tonsillectomy. The results demonstrated that more TAU patients opted for non‐surgical management versus the DA group. Thus, suggesting health professionals' implicit bias in the discussion around treatment options may directly impact treatment choice. Interestingly, this study was performed prior to the publication of the NATTINA trial, which may now influence the surgeon's approach involved in the trial, favouring tonsillectomy over non‐surgical.

More participants chose tonsillectomy within the DA group, suggesting that a DA may limit the impact of such bias. The feedback further supported this, with a common theme that participants within the DA group felt they could make an informed choice when selecting which treatment option was best for them.

### 5.1 Strengths and Limitations

One limitation of the study was contacting participants. The aim for contact at baseline was within 1 week of the consultation. However, several patients experienced a delay in contact, which may have affected their responses to the questionnaires and feedback. Furthermore, there was a disparity in the contact time between surgical and non‐surgical groups, with surgical groups being contacted later on average. Once again, this may have impacted the participants' responses.

The small sample size may not have represented the overall population. Losing several patients during follow‐up may have exacerbated this, skewing the results. Therefore, expanding this study to increase the number of participants would be advisable.

Having a single ENT consultant participate in this study was previously viewed as a strength, reducing the risk of clinician effect. However, having a single consultant explain the treatment options may have limited the range of how these treatment options may have been delivered. *The feedback demonstrated extremely positive feedback regarding the consultant*'*s technique in explaining each treatment option*. Each physician has a different approach to how they deliver information. *Unfortunately*, *the level at which the consultant delivered this information may not translate to current practice where consultations for tonsillectomy are often non*‐*consultant led*, *commonly within primary care*. *Therefore*, *the study should be repeated in other settings*, *such as primary care*. *This would help validate the efficacy of the DA and provide insights into its utility early in the decision‐making process prior to referral to ENT*.

## 6 Conclusion

The DA is an acceptable and useful tool that could be readily incorporated into a patient pathway for recurrent tonsillitis, possibly in primary care, to aid patients in making a decision while eliminating physician implicit bias. However, preliminary qualitative evidence from this pilot study does not suggest that including the DA improves the quality of decision‐making. *The study should be repeated with a larger population size and in another setting*, *such as primary care*, *to validate the findings and explore its applicability in a broader healthcare context*.

## Author Contributions

**Callum Hill:** data analysis, manuscript preparation, submission. **Kim Ah‐See:** lead Surgeon, concept, data collection, approval of final draft for submission. **Helen Moffat:** concept.

## Ethics Statement

The study was conducted in accordance with the principles of good research practice (GRP). In addition to Sponsorship approval, a favourable ethical opinion was obtained from the NHS Research Ethics Committee (REC reference: 18/ES/0098).

## Consent

The authors can confirm that written informed consent has been obtained from the involved patients and, they have given approval for this information to be published.

## Conflicts of Interest

The authors declare no conflicts of interest.

### Peer Review

The peer review history for this article is available at https://www.webofscience.com/api/gateway/wos/peer‐review/10.1111/coa.14278.

## Supporting information


**Figure S1.** Adult Tonsillectomy Decision Aid.

**Table S1.** Median values for each Shared tool for decision aid and treatment as usual both at baseline and at follow‐up.

**Table S2.** Table outlining Tonsillectomy Outcome Inventory for all patients at baseline.

## Data Availability

The data that support the findings of this study are available from the corresponding author upon reasonable request.
