# Supplementary figures and images for: Piloting of a Decision Aid for Recurrent Tonsillitis
Source: Clin Otolaryngol. 2025 Jan 16;50(3):500–6. doi: 10.1111/coa.14278 (PMC11975154; doi:10.1111/coa.14278)

Supplementary figure 1: Adult Tonsillectomy Decision Aid
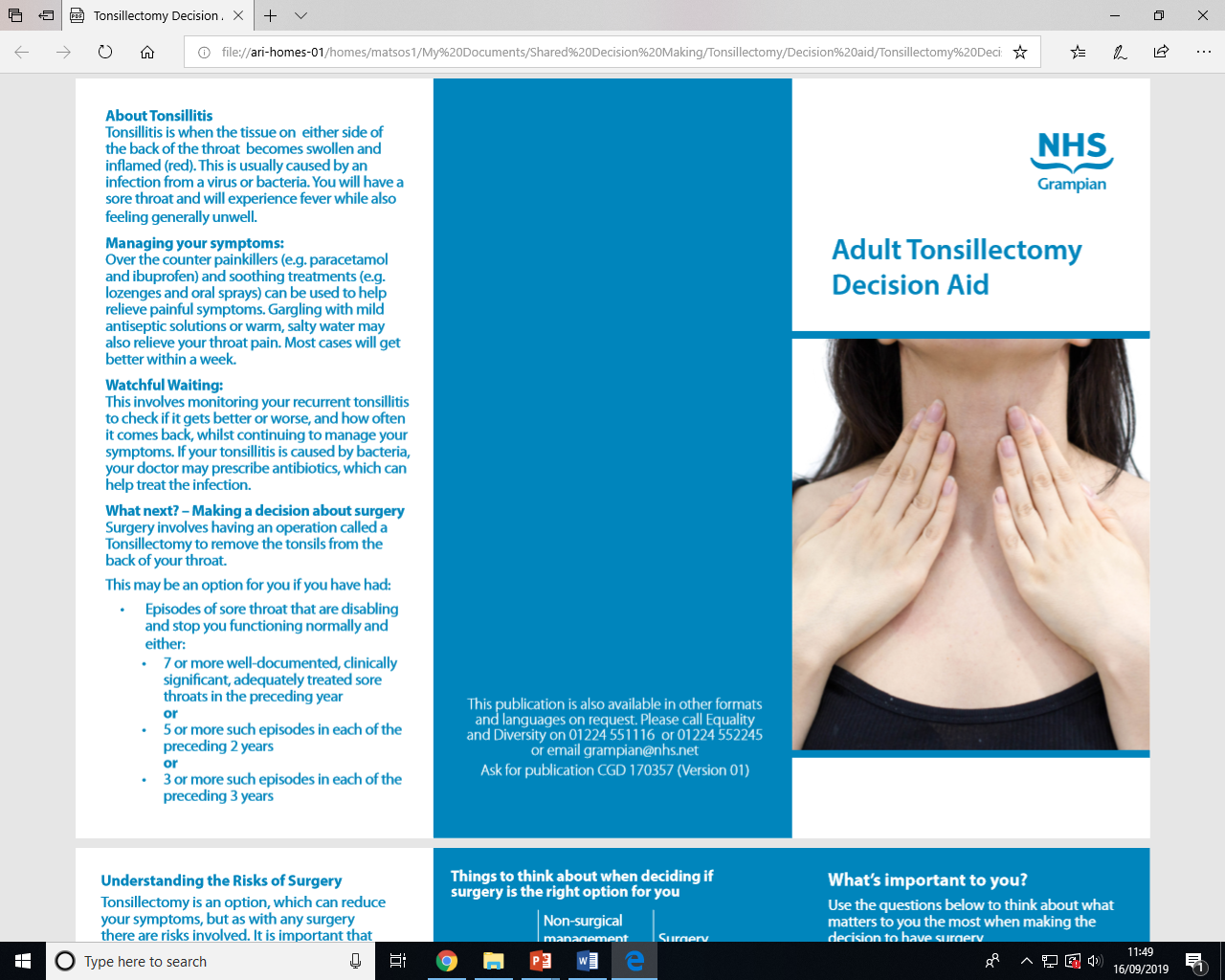


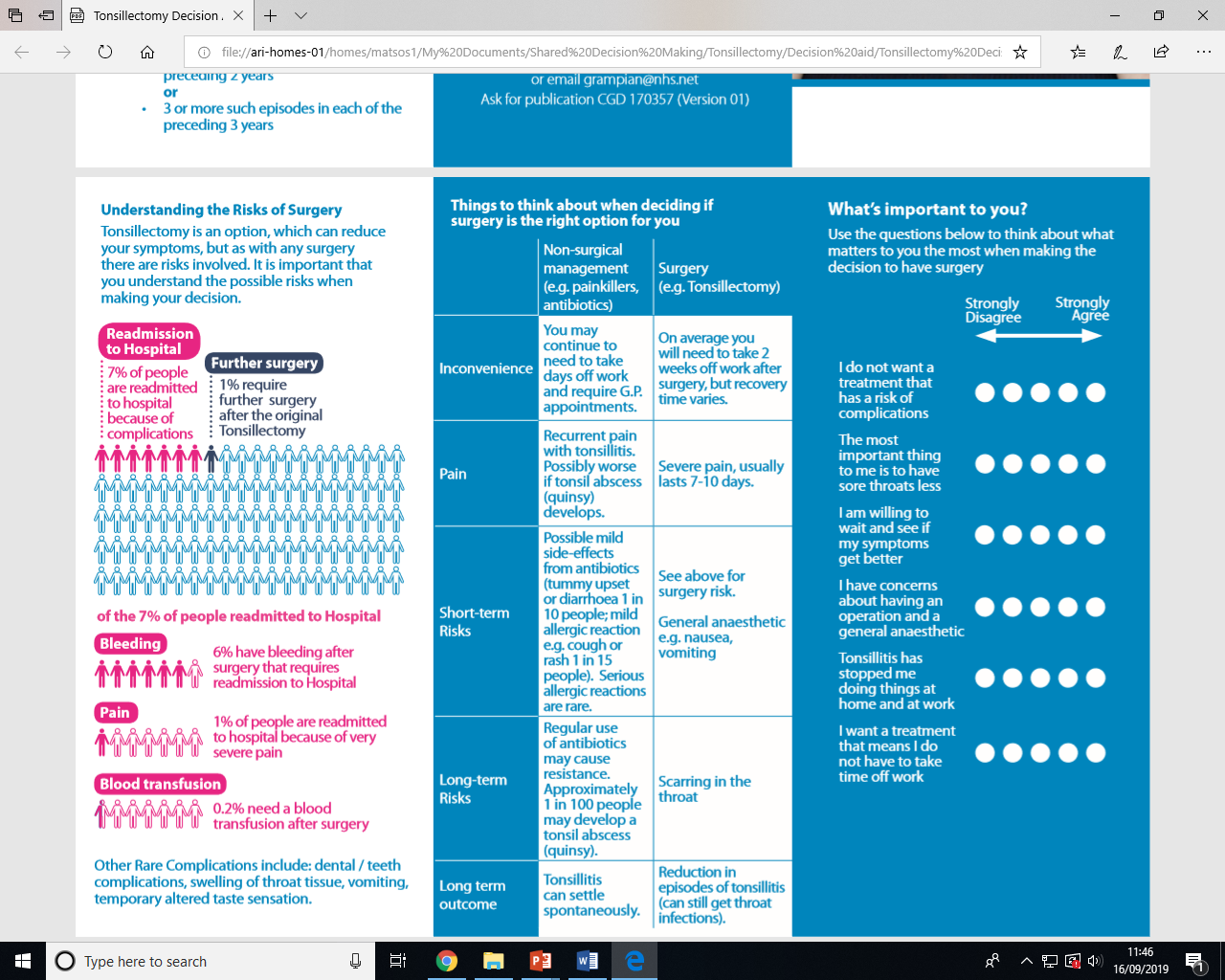

Supplement: Supplementary file 1 — Figure S1. Adult Tonsillectomy Decision Aid. [file COA-50-500-s002.docx]
