# Supplementary material for: Piloting of a Decision Aid for Recurrent Tonsillitis
Source: Clin Otolaryngol. 2025 Jan 16;50(3):500–6. doi: 10.1111/coa.14278 (PMC11975154; doi:10.1111/coa.14278)
Supplement: Supplementary file 2 — Table S1. Median values for each Shared tool for decision aid and treatment as usual both at baseline and at follow‐up. [file COA-50-500-s001.docx]

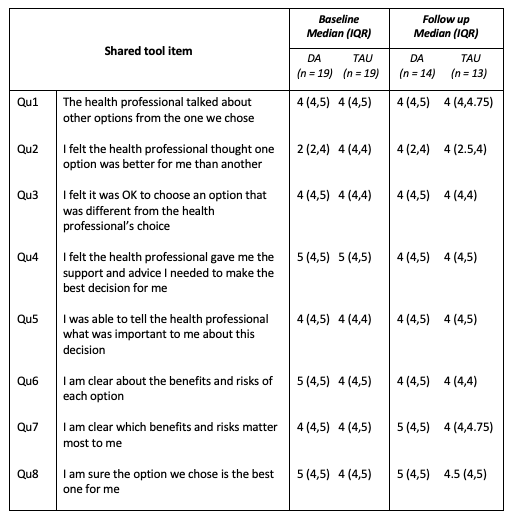


**Supplementary Table 1**. Median values for each shared tool for decision aid and treatment as usual both at baseline and at follow-up.

Note: [1=Strongly agree, 2 = Disagree, 3 = Neutral, 4 = Agree, 5 = Strongly agree]

Abbreviation: DA, decision aid; TAU, treatment as usual
