# Supplementary material for: Piloting of a Decision Aid for Recurrent Tonsillitis
Source: Clin Otolaryngol. 2025 Jan 16;50(3):500–6. doi: 10.1111/coa.14278 (PMC11975154; doi:10.1111/coa.14278)
Supplement: Supplementary file 3 — Table S2. Table outlining Tonsillectomy Outcome Inventory for all patients at baseline. [file COA-50-500-s003.docx]

Supplementary table 2. Table outlining Tonsillectomy Outcome Inventory for all patients at baseline.

| **Patient Number** | **Group** | **Tonsillectomy Outcome Inventory** | | | | | |
| --- | --- | --- | --- | --- | --- | --- | --- |
|  |  |  |  |  |  |  |  |
|  |  | *Total* | *Throat Discomfort* | *Geneal health* | *Resources* | *Social Psychological restrictions* | *Other* |
| P1 | TAU | 72.86 | 90 | 70 | 75 | 55 | / |
| P2 | TAU | 58.57 | 45 | 40 | 70 | 70 | / |
| P3 | DA | 62.86 | 60 | 70 | 55 | 70 | / |
| P4 | DA | 75.71 | 80 | 100 | 65 | 70 | / |
| P5 | DA | 78.57 | 80 | 90 | 80 | 70 | / |
| P6 | DA | 65.71 | 85 | 70 | 55 | 55 | / |
| P7 | TAU | 60 | 40 | 80 | 75 | 55 | / |
| P8 | TAU | 50 | 65 | 70 | 45 | 30 | / |
| P9 | DA | 54.29 | 35 | 90 | 65 | 45 | / |
| P10 | TAU | 34.29 | 35 | 70 | 50 | 0 | / |
| P11 | DA | 50 | 65 | 50 | 25 | 60 | / |
| P12 | DA | 64.29 | 70 | 80 | 65 | 50 | / |
| P13 | TAU | 77.14 | 70 | 90 | 90 | 65 | / |
| P14 | TAU | 51.43 | 75 | 80 | 50 | 15 | Sleeping 5 |
| P15 | TAU | 64.29 | 85 | 80 | 45 | 55 | / |
| P16 | TAU | 10 | 5 | 0 | 15 | 15 | Interferes with asthma |
| P17 | TAU | 61.43 | 75 | 60 | 50 | 60 | / |
| P18 | DA | 51.43 | 50 | 80 | 50 | 40 | / |
| P19 | DA | 60 | 55 | 80 | 65 | 50 | / |
| P20 | TAU | 68.57 | 90 | 60 | 40 | 80 | Ability to speak 3 |
| P21 | DA | 65.71 | 80 | 60 | 60 | 60 | Ear pain, fainting |
| P22 | TAU | 64.29 | 75 | 80 | 55 | 55 | / |
| P23 | DA | 71.43 | 65 | 100 | 70 | 65 | / |
| P24 | DA | 85.71 | 70 | 100 | 95 | 85 | / |
| P25 | DA | 72.86 | 80 | 90 | 80 | 50 | / |
| P26 | TAU | 30 | 45 | 30 | 15 | 30 | / |
| P27 | TAU | 54.29 | 75 | 40 | 50 | 45 | / |
| P28 | DA | 57.14 | 70 | 50 | 50 | 55 | / |
| P29 | DA | 62.86 | 65 | 90 | 60 | 50 | / |
| P30 | DA | 41.43 | 65 | 50 | 35 | 20 | / |
| P31 | TAU | 74.29 | 90 | 80 | 90 | 40 | / |
| P32 | TAU | 32.86 | 35 | 20 | 50 | 20 | / |
| P33 | DA | 47.14 | 40 | 60 | 65 | 30 | / |
| P34 | DA | 61.43 | 70 | 90 | 65 | 35 | Ability to look after my children |
| P35 | TAU | 61.43 | 65 | 70 | 55 | 60 | / |
| P36 | TAU | 51.43 | 80 | 40 | 50 | 30 | / |
| P37 | TAU | 31.43 | 45 | 30 | 50 | 0 | / |
| P38 | DA | 47.14 | 60 | 60 | 30 | 45 | / |
